# The Effect of Botanical Pesticides Azadirachtin, Celangulin, and Veratramine Exposure on an Invertebrate Species *Solenopsis invicta* (Hymenoptera: Formicidae)

**DOI:** 10.3390/toxins16010006

**Published:** 2023-12-20

**Authors:** Yuling Liang, Mingrong Liang, Huimei Chen, Jingxin Hong, Yunbo Song, Kuo Yue, Yongyue Lu

**Affiliations:** 1 Red Imported Fire Ant Research Center, South China Agricultural University, Guangzhou 510642, China; liangyuling@stu.scau.edu.cn (Y.L.); liangmr0321@connect.hku.hk (M.L.);; 2 Insect Biodiversity and Biogeography Laboratory, School of Biological Sciences, The University of Hong Kong, Pok Fu Lam Road, Hong Kong SAR, China

**Keywords:** botanical pesticides, azadirachtin, celangulin, veratramine, toxicity, red imported fire ant, nontarget organism

## Abstract

The injudicious and excessive use of synthetic pesticides has deleterious effects on humans, ecosystems, and biodiversity. As an alternative to traditional crop-protection methods, botanical pesticides are gaining importance. In this research endeavor, we examined the contact toxicity, knockdown time, lethal time, and toxicity horizontal transmission of three natural pesticides from plants (azadirachtin, celangulin, and veratramine) on red imported fire ants (RIFA; *Solenopsis invicta*). Our research findings indicated that azadirachtin and celangulin exhibited relatively high toxicity, with median lethal dose (LD_50_) values of 0.200 and 0.046 ng/ant, respectively, whereas veratramine exhibited an LD_50_ value of 544.610 ng/ant for large workers of *S. invicta* at 24 h post-treatment. Upon treatment with 0.125 mg/L, the (median lethal time) LT_50_ values of azadirachtin and celangulin were determined to be 60.410 and 9.905 h, respectively. For veratramine, an LT_50_ value of 46.967 h was achieved after being tested with 200 mg/L. Remarkably, azadirachtin and celangulin were found to exhibit high horizontal transfer among RIFA, with high secondary mortality (100%) and tertiary mortalities (>61%) after 48 h of treatment with 250 mg/L, as well as with their dust formulations for 72 h. However, veratramine did not exhibit significant toxicity or horizontal transfer effects on RIFA, even at high concentrations. These findings suggest that azadirachtin and celangulin are likely to have a highly prominent potential in the management of *S. invicta*.

## 1. Introduction

In response to the escalating demand for food to sustain a growing global population, synthetic chemicals have been developed and utilized as a rapid and effective approach to control crop pests and diseases [1]. However, the problems associated with synthetic pesticides and their residues have necessitated the development of effective biodegradable pesticides with enhanced selectivity [2]. Many synthetic pesticides are not readily biodegradable, leading to their accumulation in the environment and causing contamination of soil and groundwater, as well as contributing to ozone depletion [3,4,5].

Moreover, the excessive and improper use of synthetic pesticides can have detrimental effects on nontarget organisms, leading to toxicity, and can also negatively impact biodiversity [4]. Most importantly, certain ecologically sensitive areas, such as organic plantations, nature reserves, and wetlands, prohibit the use of synthetic chemicals [6].

Botanical pesticides have emerged as prominent alternatives to conventional synthetic chemical pesticides, offering several advantages [1,7,8,9]. These alternatives are cost effectiveness, biodegradability, possessing multiple modes of action, being readily accessible, and exhibiting low toxicity toward nontarget organisms [3,10,11,12]. Essential oils derived from plants and similar products typically demonstrate acute toxicity levels exceeding 2 g/kg via ingestion and contact, while exhibiting nontoxicity to mammals, birds, and fish [12]. Therefore, botanical pesticides are considered highly promising materials for pest control in ecological areas.

Currently, the most promising botanical insecticide for integrated pest management is azadirachtin, which exhibits a broad range of bioactivities. These include antifeeding, growth inhibition, larval toxicity, and oviposition deterrent effects on Lepidoptera pests [13,14], as well as nematicidal properties [12]. The highest insecticidal activity has been identified in the seeds and leaves of the neem plant [15,16]. Furthermore, studies have shown that azadirachtin disrupts hormonal balance and inhibits the activity of acetylcholinesterase (AChE) [12], making it highly effective against sucking pests, such as leafhoppers, aphids, and whiteflies [17,18,19].

Celangulins, a group of bioactive compounds derived from the Chinese bittersweet plant *Celastrus angulatus* (Celastraceae), have been found to possess antifeedant, narcotic, and insecticidal properties against various pests, including the rice-plant skipper, cotton semilooper, cotton-leaf roller, and cabbage caterpillar. These effects are attributed to the disruption of the intestinal wall cell membrane and organelle membrane in Lepidoptera larvae [20,21,22,23,24]. Specifically, celangulin IV and V exhibit targeting of the neuromuscular synapse and digestive system, inducing narcosis by blocking the neuromuscular junction and causing cell death in the midgut through organelle damage [9,20,24,25]. In a study conducted by Wei et al. [26], it was demonstrated that the mortality rates of *Ectropis obipua hypulina* second instar larvae were 98.67%, 85.33%, and 73.33% after a 3-day exposure to tea leaves treated with 0.2% celangulin at concentrations of 500, 1000, and 1500 times, respectively. Moreover, Qi et al. [27,28] reported that the novel botanical insecticide celangulins exhibited low toxicity towards nontarget organisms. These findings confirm that celangulins can be classified as an environmentally friendly pesticide.

Veratramine, an alkaloid derived from traditional medicinal herbs such as Veratrum and Fritillaria species, exhibits contact-killing, stomach toxicity, anti-inflammatory, antihypotensive, antioxidative, and antifungal activities [29,30]. Previous research has demonstrated that veratramine exhibits effective control of sucking insects, including aphids and mites [31,32], and shows high lethal activity against *Apolygus lucorum* [33]. These findings suggest that botanical pesticides may represent promising alternatives to synthetic pesticides for effective pest control.

The red imported fire ant (RIFA), *Solenopsis invicta* Buren, is a widely distributed invasive ant species [34] and has been extensively studied as one of the most well-researched ant species [35,36]. Originally native to South America, *S. invicta* was inadvertently introduced into the United States in the 1930s [37]. Since then, this species has rapidly expanded its range across four continents (North America, Oceania, Africa, and Asia) and numerous islands, owing to its remarkable reproductive capacity and dispersal abilities [38,39]. Currently, *S. invicta* has become one of the most prevalent ant species in South China [40,41,42]. Although RIFA has been acknowledged by certain researchers as a valuable participant in biological pest control across various agricultural domains, such as cotton fields [43,44], sugarcane plantations [45,46,47], soybean crops [48], and greenhouse cultivation [49], the detrimental consequences it imposes often outweigh its benefits. This is primarily due to the fact that RIFA can engender substantial hazards to human health, public safety, agriculture, economy, and ecology in regions where it has been introduced [34,50]. The invasion of RIFA has been observed to cause detrimental ecological consequences across various habitats, leading to disturbances in arthropod community structure and a decline in the diversity and abundance of indigenous ant species [41]. Research findings indicate that approximately one-third of individuals residing in RIFA-infested areas in China have reported experiencing ant stings, with approximately 10% of them suffering from severe hypersensitivity reactions [51].

Multiple studies have demonstrated that exposure to chemical synthetic pesticides can have adverse effects on ant species. For example, the use of imidacloprid has been shown to reduce the aggressiveness of two European ant species, *Lasius niger* and *L. flavus*, leading to potential changes in established dominance hierarchies among ant populations [52]. In addition, research has shown that field-relevant levels of imidacloprid can inhibit the digging and feeding behaviors of workers and reduce the fitness of incipient colonies of RIFA [53,54]. Exposure to sulfoxaflor has also produced significant negative effects on *S. invicta* colony growth, with cumulative colony weight losses of 83.36% and 100.00% after treatment with 1 μg/mL and 2 μg/mL, respectively [36]. Moreover, numerous investigations have established that fipronil and indoxacarb are the predominant active ingredients employed in baits utilized for the management of RIFA in the field [55,56,57]. However, the prolonged and improper application of these restricted chemical insecticides in the control of RIFA may foster the development of resistance or tolerance. In this regard, Zhang et al. [58] uncovered a significant 36.4-fold surge in cytochrome P450 detoxification enzyme genes in worker ants of RIFA subsequent to treatment with fipronil. Additionally, Xiong et al. [55] elucidated that the activity of cytochrome P450 in larvae was approximately 24 times greater than that in workers. Additionally, adult workers displayed a fourfold increase in fipronil-induced cytochrome P450 activity compared to individuals from other adult castes subsequent to fipronil treatment. In the case of indoxacarb, Siddiqui et al. [59] observed substantial differential expression in a majority of genes associated with detoxification enzymes, with 100 genes being significantly upregulated and 58 genes significantly downregulated in red imported fire ants following exposure to indoxacarb. In light of the potential emergence of resistance resulting from the limited repertoire of chemical pesticides utilized against *S. invicta*, there is a pressing need to expeditiously assess novel plant-based compounds for the purpose of achieving effective control over the RIFA population.

Despite the extensive testing of azadirachtin, celangulin, and veratramine on Lepidoptera, Hemiptera, and Diptera pests [11,14,21], their contact toxicity to pest ant species, specifically the red imported fire ant, has not been assessed. Therefore, this study aims to evaluate the contact toxicity, knockdown time, lethal time, and horizontal transmission toxicity of these three natural plant pesticides on RIFA.

## 2. Results

### 2.1. Contact Toxicity of Azadirachtin, Celangulin, and Veratramine on Workers

The lethal dose (LD) values of azadirachtin, celangulin, and veratramine are shown in Table 1. Following a post-treatment duration of 12, 24, 36, and 48 h, the LD_50_ values of azadirachtin for large workers of *S. invicta* were 0.369, 0.200, 0.160, and 0.149 ng/ant, respectively, while the LD_95_ values were 1.571, 0.610, 0.454, and 0.388 ng/ant, respectively. Similarly, the LD_50_ values of celangulin for the same group of ants were 0.086, 0.046, 0.039, and 0.035 ng/ant, with corresponding LD_95_ values of 0.351, 0.119, 0.106, and 0.093 ng/ant following post-treatment durations of 12, 24, 36, and 48 h. Lastly, the LD_50_ values of veratramine for large workers of *S. invicta* were 544.610, 234.074, and 160.470 ng/ant, while the LD_95_ values were 9041.335, 2588.682, and 1634.820 ng/ant following post-treatment durations of 24, 36, and 48 h.

The lethal time (LT) values of azadirachtin, celangulin, and veratramine are presented in Table 2. RIFA exhibited shorter knockdown times as the concentrations of these three botanicals increased. For azadirachtin, the LT_50_ and LT_95_ values were 7.709 and 15.856 h, 8.693 and 31.841 h, 16.839 and 121.642 h, and 60.410 and 403.803 h, respectively, when treated with concentrations of 1, 0.5, 0.25, and 0.125 mg/L (Table 2). In the case of celangulin, the LT_50_ and LT_95_ values were 9.905 and 25.227 h, 14.433 and 194.821 h, and 53.918 and 385.491 h, respectively, when treated with concentrations of 0.125, 0.063, and 0.031 mg/L. For veratramine, the LT_50_ and LT_95_ values were 24.407 and 74.021 h, and 46.967 and 169.399 h, respectively, when treated with concentrations of 500 and 200 mg/L (Table 2).

### 2.2. Knockdown Time of Azadirachtin, Celangulin, and Veratramine on Workers

The knockdown times (KT) of azadirachtin and celangulin are provided in Table 3. It was observed that higher concentrations of these botanicals resulted in shorter knockdown times in RIFA. Specifically, for azadirachtin, the KT_25_ values of 500, 100, and 50 mg/L were measured as 4.937, 8.811, and 10.774 min, respectively. The KT_50_ values were 5.963, 11.187, and 14.581 min, respectively, and the KT_95_ values were 9.449, 20.027, and 30.493 min, respectively. Similarly, for celangulin, the KT_25_ values of 500, 100, and 50 mg/L were determined to be 4.937, 8.811, and 10.774 min, respectively. The KT_50_ values were 5.963, 11.187, and 14.581 min, respectively, and the KT_95_ values were 9.449, 20.027, and 30.493 min, respectively. In contrast, veratramine did not exhibit any knockdown effect on large workers within one hour. These findings suggest that azadirachtin and celangulin demonstrate significant knockdown effects on RIFA.

### 2.3. Effect of Soil Contaminated with Azadirachtin, Celangulin, and Veratramine on Workers

**Figure 1:**
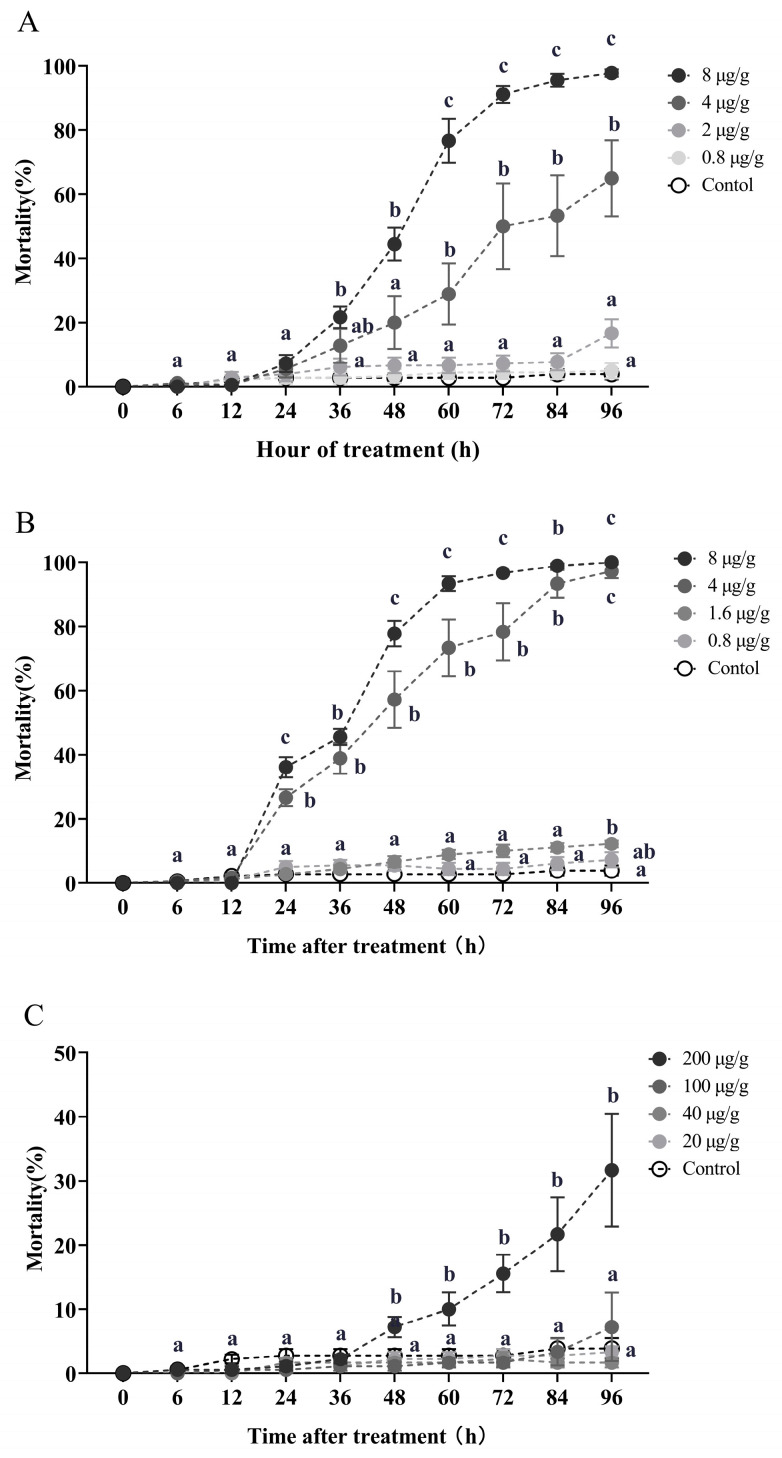
The impact of soil contaminated with azadirachtin (**A**), celangulin (**B**), and veratramine (**C**) on workers. Data in the figure are the mean ± standard error. Identical letters in the figure indicate no significant difference (*p* > 0.05) by the Kruskal–Wallis test.

The worker mortality was performed in Figure 1 resulting from exposure to azadirachtin, celangulin, and veratramine at varying concentrations of contaminated soil for different durations. For azadirachtin, worker mortality reached 44.44% and 20% after exposure to 8 and 4 μg/g concentration toxic soil for 48 h, respectively. Mortality rates increased to 91.11% and 50% at 72 h and reached 97.78% and 65% at 96 h, respectively (Kruskal–Wallis test, 48 h: *χ*^2^ = 17.065, *df* = 4, *p* = 0.002; 72 h: *χ*^2^ = 20.361, *df* = 4, *p* < 0.001; 96 h: *χ*^2^ = 23.205, *df* = 4, *p* < 0.001; Figure 1A). Similarly, for celangulin, worker mortality was 36.11% and 26.67% at 24 h for 8 and 4 μg/g concentration toxic soil, respectively, and reached 77.78% and 57.22% at 48 h and 96.67% and 78.33% at 72 h, ultimately causing 100% and 97.22% mortality at 96 h, respectively (Kruskal–Wallis test, 48 h: *χ*^2^ = 22.923, *df* = 4, *p* < 0.001; 72 h: *χ*^2^ = 24.093, *df* = 4, *p* < 0.001; 96 h: *χ*^2^ = 24.697, *df* = 4, *p* < 0.001; Figure 1B). Conversely, exposure to 200 μg/g concentration of veratramine soil caused 15.56% and 31.67% mortality at 72 and 96 h, respectively (Kruskal–Wallis test, 72 h: *χ*^2^ = 15.193, *df* = 4, *p* = 0.004; 96 h: *χ*^2^ = 13.585, *df* = 4, *p* = 0.009; Figure 1C). The worker mortalities were all below 7.5% when exposed to 100, 40, and 20 μg/g concentrations of veratramine soil for 96 h (Figure 1C).

When treated with 8 μg/g contaminated soil, celangulin demonstrated a faster killing efficacy compared to azadirachtin, as indicated by a shorter LT_50_ (32.734 h) and LT_95_ (67.979 h) when compared to azadirachtin (LT_50_ of 46.256 h and LT_95_ of 86.469 h) (Table A1). Conversely, veratramine exhibited a slower lethal time, with an LT_50_ of 152.972 h and an LT_95_ of 588.644 h, when treated with the same concentration of 8 μg/g soil (Table A1).

### 2.4. Horizontal Toxicity Transfer of Azadirachtin, Celangulin, and Veratramine on Workers

**Figure 2:**
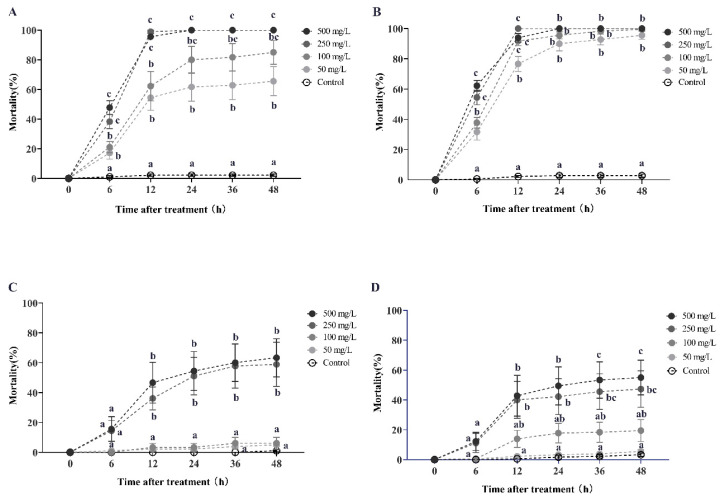
This study examines the horizontal transfer of azadirachtin toxicity in red imported fire ants. The data presented in the figure are expressed as the mean ± standard error. (**A**,**C**) represent the first-round and second-round toxicity transfer, respectively, with a donor-workers to the recipient-workers ratio of 1:10. Similarly, (**B**,**D**) represent the first-round and second-round toxicity transfer with a donor-workers to recipient-workers ratio of 1:5. In the figure, identical letters indicate no significant difference (*p* > 0.05) as determined by the Kruskal–Wallis test.

Azadirachtin solution demonstrated a high degree of horizontal toxicity transfer in RIFA, resulting in increased mortality. Specifically, a higher donor-to-recipient worker ratio led to greater mortality in RIFA (Figure 2). During the initial round of horizontal transfer, the secondary mortality of recipient workers (with a donor-workers to the recipient-workers ratio of 1:10) progressively increased with treatment duration and concentration. After 6 h of exposure to azadirachtin solution at concentrations of 500, 250, 100, and 50 mg/L, the secondary mortality rates of recipient workers were 47.78%, 38.33%, 21.11%, and 17.22%, respectively. These rates increased to 95.56%, 98.89%, 62.22%, and 54.44% at 12 h, and ultimately reached 100%, 100%, 85%, and 65.55% at 48 h. Statistical analysis using the Kruskal–Wallis test confirmed the significance of the results at each time point (6 h: *χ*^2^ = 22.651, *df* = 4, *p* < 0.001; 12 h: *χ*^2^ = 25.935, *df* = 4, *p* < 0.001; 48 h: *χ*^2^ = 26.236, *df* = 4, *p* < 0.001; Figure 2A). Similarly, when the donor-workers to recipient-workers ratio was 1:5, the secondary mortality rates were 62.22%, 54.44%, and 37.78% after 6 h of treatment with 500, 250, 100, and 50 mg/L azadirachtin solution, respectively. These rates increased to 93.89%, 100%, 91.67%, and 76.67% at 12 h, and reached 100%, 100%, 99.45%, and 95.56% at 48 h. The Kruskal–Wallis test confirmed the statistical significance of these findings as well (6 h: *χ*^2^ = 23.404, *df* = 4, *p* < 0.001; 12 h: *χ*^2^ = 23.670, *df* = 4, *p* < 0.001; 48 h: *χ*^2^ = 22.628, *df* = 4, *p* < 0.001; Figure 2B). However, a high donor-to-recipient worker ratio did not result in a significantly shorter lethal time for RIFA when exposed to high concentrations of azadirachtin at 500 mg/L during the first-round toxicity transmission. This was evidenced by the LT_50_ values of 6.139 h and 5.113 h, and LT_95_ values of 11.729 h and 12.294 h, for donor-to-recipient ratios of 1:10 and 1:5, respectively (Table A2).

During the second round of horizontal transfer, the tertiary mortality of recipient workers (with a donor-workers to the recipient-workers ratio of 1:10) increased with the concentration and duration of exposure to azadirachtin solution. After 12 h of treatment with 500 and 250 mg/L azadirachtin, the tertiary mortality rates were 36.11% and 46.67%, respectively, as confirmed by the Kruskal–Wallis test (*χ*^2^ = 23.419, *df* = 4, *p* < 0.001; Figure 2C). At 24 h, the tertiary mortality rates of recipient workers further increased to 51.11% and 54.45% (*χ*^2^ = 23.836, *df* = 4, *p* < 0.001), respectively, and were both higher than 61% at 48 h (*χ*^2^ = 22.971, *df* = 4, *p* < 0.001). However, when treated with 100 and 50 mg/L azadirachtin, the tertiary mortality of recipient workers was lower than 5% at 48 h, which was not significantly different from the control group. 

Similarly, when the donor-workers to recipient-workers ratio was 1:5, the tertiary mortality rates were 42.78%, 40%, and 13.89% after 12 h of treatment with 500, 250, and 100 mg/L azadirachtin, respectively (Kruskal–Wallis test, *χ*^2^ = 17.708, *df* = 4, *p* = 0.001; Figure 2D). At 48 h, the tertiary mortality rates further increased to 55%, 47.22%, and 19.44%, respectively (Kruskal–Wallis test, *χ*^2^ = 19.060, *df* = 4, *p* = 0.001). During this round of toxicity transmission, azadirachtin exhibited a slower lethal time on RIFA, as indicated by the LT_50_ values of 381.461 h and 614.665 h, respectively, when treated with concentrations of 500 and 250 mg/L, for a donor-to-recipient ratio of 1:10 (Table A3).

**Figure 3:**
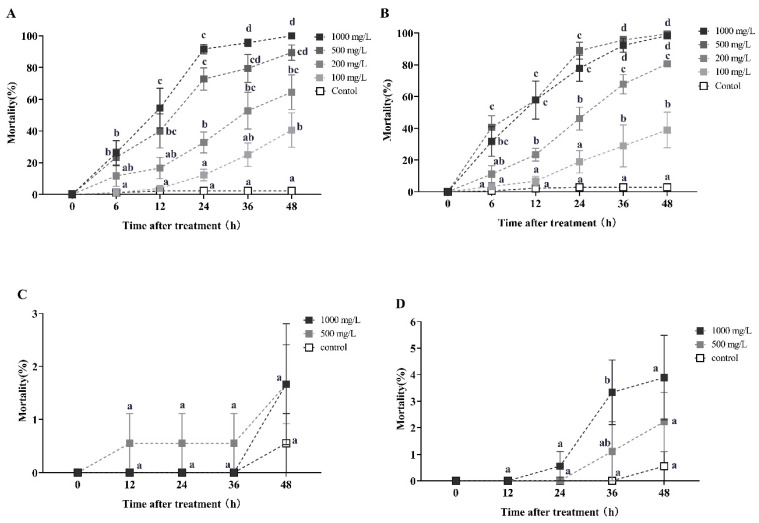
This study investigates the horizontal transfer of celangulin toxicity in red imported fire ants. The data presented in the figure are expressed as the mean ± standard error. (**A**,**C**) represent the first-round and second-round toxicity transfer, respectively, with a donor-workers to the recipient-workers ratio of 1:10. Similarly, (**B**,**D**) represent the first-round and second-round toxicity transfer with a donor-workers to recipient-workers ratio of 1:5. In the figure, identical letters indicate no significant difference (*p* > 0.05) as determined by the Kruskal–Wallis test.

Celangulin solution demonstrated a significant horizontal transfer effect on recipient workers only during the first round of transmission (Figure 3). After 12 h of treatment with 1000, 500, and 200 mg/L celangulin solutions, the secondary mortality rates of recipient workers (with a donor-workers to the recipient-workers ratio of 1:10) were 54.45%, 40%, and 16.67%, respectively. These rates increased to 91.67%, 72.78%, and 32.78% at 24 h, and ultimately reached 100%, 89.45%, and 64.45% at 48 h. The Kruskal–Wallis test confirmed the statistical significance of these findings at each time point (12 h: *χ*^2^ = 20.400, *df* = 4, *p* < 0.001; 24 h: *χ*^2^ = 25.732, *df* = 4, *p* < 0.001; 48 h: *χ*^2^ = 24.579, *df* = 4, *p* < 0.001; Figure 3A). Similarly, when the donor-workers to recipient-workers ratio was 1:5, the secondary mortality rates were 57.75%, 57.78%, and 23.33% after 12 h of treatment with 1000, 500, and 200 mg/L celangulin solution, respectively. These rates increased to 77.78%, 88.89%, and 46.11% at 24 h, and ultimately reached 98.33%, 100%, and 80.56% at 48 h (Kruskal–Wallis test, 12 h: *χ*^2^ = 22.680, *df* = 4, *p* < 0.001; 24 h: *χ*^2^ = 24.678, *df* = 4, *p* < 0.001; 48 h: *χ*^2^ = 27.285, *df* = 4, *p* < 0.001; Figure 3B). Furthermore, a high donor-to-recipient worker ratio resulted in a significantly shorter lethal time for RIFA, as evidenced by the LT_50_ values of 13.900 h and 8.369 h, and LT_95_ values of 78.374 h and 34.872 h, for donor-to-recipient ratios of 1:10 and 1:5, respectively (Table A2).

However, celangulin exhibited an ineffective horizontal transfer effect in the second round of transmission, with the tertiary mortality rates of recipient workers (with a donor-workers to the recipient-workers ratio of 1:10 and 1:5) being lower than 4% after 48 h of treatment with 1000 mg/L dosage (Figure 3C,D).

**Figure 4:**
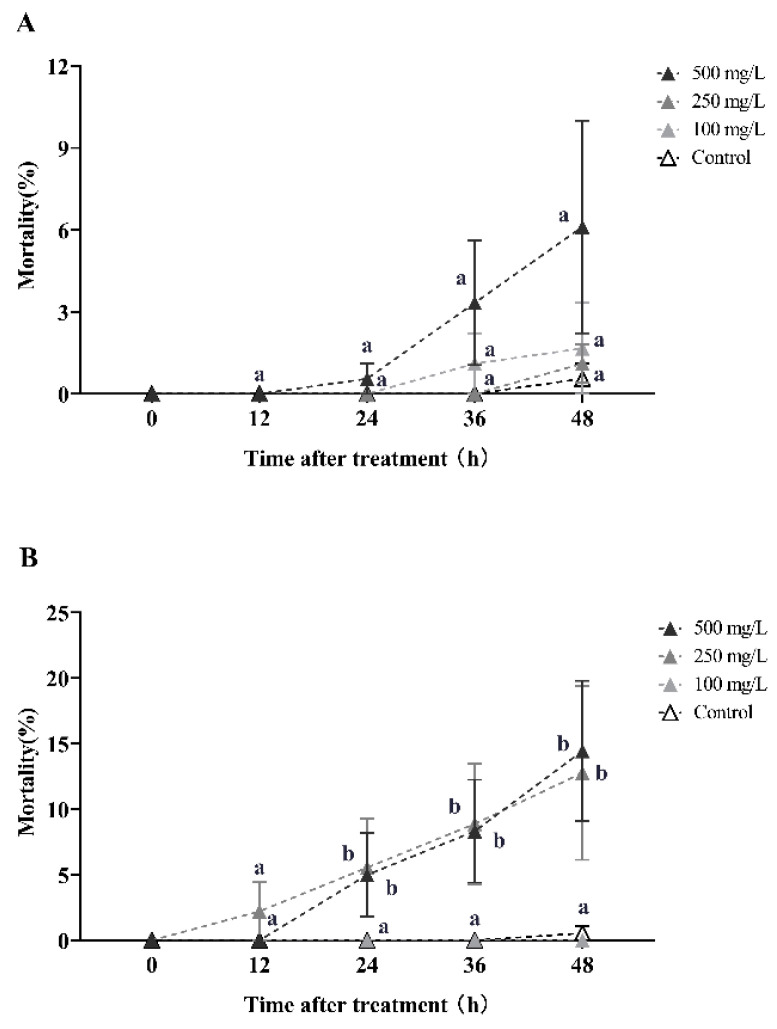
This study examines the horizontal transfer of veratramine toxicity in red imported fire ants. The data presented in the figure are expressed as the mean ± standard error. (**A**,**B**) represent the first-round toxicity transfer with donor-workers to recipient-workers ratios of 1:10 and 1:5, respectively. In the figure, identical letters indicate no significant difference (*p* > 0.05) as determined by the Kruskal–Wallis test.

Similarly, veratramine exhibited an ineffective horizontal transfer effect in the first round of transmission, with the tertiary mortality rates of recipient workers (with a donor-workers to the recipient-workers ratio of 1:10 and 1:5) being lower than 14.44% after 48 h of treatment with 500 mg/L concentration (Figure 4).

### 2.5. Horizontal Toxicity Transfer of Azadirachtin, Celangulin, and Veratramine Dust on Workers

**Figure 5:**
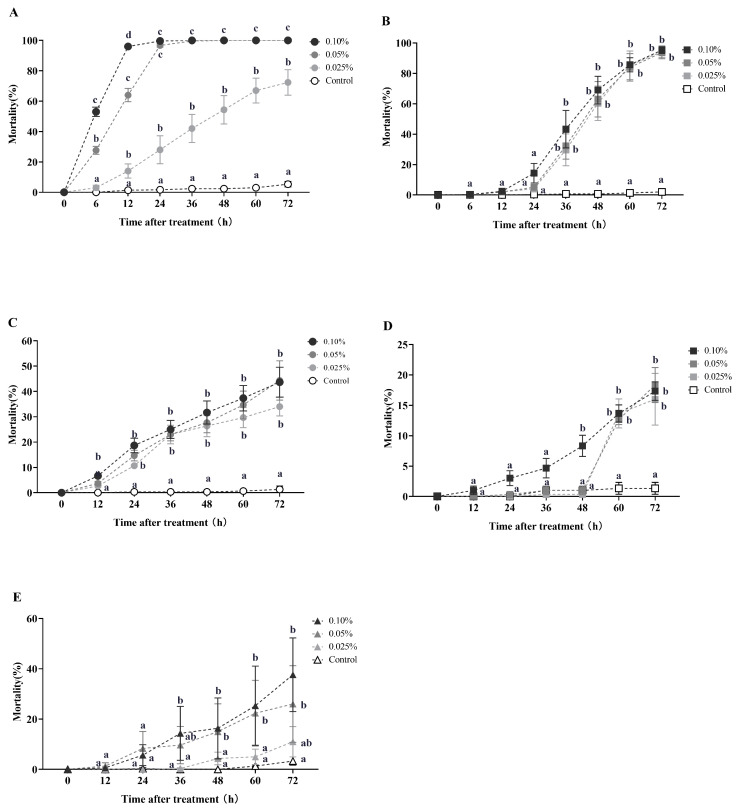
This study investigates the horizontal toxicity transfer of azadirachtin, celangulin, and veratramine dust on red imported fire ants. The data presented in the figure are expressed as the mean ± standard error. (**A**,**C**) represent the first-round and second-round toxicity transfer of azadirachtin dust on workers, respectively, with a donor-workers to recipient-workers ratio of 1:10. Similarly, (**B**,**D**) represent the first-round and second-round toxicity transfer of celangulin dust on workers, respectively, with the same donor-workers to recipient-workers ratio. (**E**) represents the first-round toxicity transfer of veratramine dust formulation on workers with a donor-workers to the recipient-workers ratio of 1:10. In the figure, identical letters indicate no significant difference (*p* > 0.05) as determined by the Kruskal–Wallis test.

Azadirachtin and celangulin dust demonstrated effective horizontal toxicity transfer in RIFA during the initial round of horizontal toxicity transmission (Figure 5). The secondary mortality of recipient workers (donor workers–recipient workers = 1:10) was 27.67% and 53% after 6 h of treatment with 0.05% and 0.10% azadirachtin dust, respectively. This mortality rate increased to 64% and 96% after 12 h, respectively. At 24 h, the secondary mortality of recipient workers reached 28%, 96.67%, and 99.67% for treatments with concentrations of 0.025%, 0.05%, and 0.10%, respectively. These rates were all significantly higher than the control group (Kruskal–Wallis test, *χ*^2^ = 21.077, *df* = 3, *p* < 0.001; Figure 5A). The secondary mortality of the recipient workers reached 72.33% at 72 h after treatment with 0.025% azadirachtin dust.

In the first round of horizontal toxicity transfer involving celangulin dust, the secondary mortality of recipient workers (donor workers–recipient workers = 1:10) was observed to be 4%, 5%, and 14.33% at 24 h post-treatment, with concentrations of 0.025%, 0.05%, and 0.10% celangulin dust, respectively. Subsequently, the secondary mortalities of recipient workers increased to 60.33%, 63%, and 69% at 48 h post-treatment, with the respective concentrations. At 72 h post-treatment, the secondary mortalities of recipient workers caused by 0.025%, 0.05%, and 0.10% celangulin dust exceeded 93%, which was significantly higher than the control group (Kruskal–Wallis test, *χ*^2^ = 14.416, *df* = 3, *p* = 0.002; Figure 5B). Interestingly, when treated with a 0.10% dust formulation, celangulin appeared to have a slower lethal time compared to azadirachtin. Celangulin demonstrated LT_50_ and LT_95_ values of 37.435 h and 79.107 h, respectively, whereas azadirachtin exhibited an LT_50_ value of 5.769 h and an LT_95_ value of 11.947 h (Table A4).

During the second round of toxicity horizontal transfer, the tertiary mortality of recipient workers (donor workers–recipient workers = 1:10) caused by 0.025%, 0.05%, and 0.10% azadirachtin dust was 26.33%, 27%, and 31.67%, respectively, 48 h post-treatment (Kruskal–Wallis test, *χ*^2^ = 13.643, *df* = 3, *p* = 0.003; Figure 5C). At 72 h post-treatment, the tertiary mortality of workers was 34%, 43.67%, and 44.33%, respectively, all of which were significantly higher than the control group (Kruskal–Wallis test, *χ*^2^ = 14.399, *df* = 3, *p* = 0.002). During this round of toxicity transmission, azadirachtin demonstrated a slower lethal time when treated with different concentrations of dust formulation. Specifically, LT_50_ values of 91.773 h, 89.958 h, and 111.736 h were observed for concentrations of 0.10%, 0.05%, and 0.25%, respectively (Table A5). 

In the second round of toxicity horizontal transfer, the tertiary mortality of recipient workers (donor workers–recipient workers = 1:10) was observed to be 16%, 17.33%, and 18.33% at 72 h post-treatment, with concentrations of 0.25%, 0.50%, and 1.00% celangulin dust, respectively. There was no significant difference in the tertiary mortality among the concentrations of 0.25%, 0.50%, and 1.00% dimefluthrin dust treatments (Figure 5D).

Regarding veratramine dust, the secondary mortality of recipient workers (donor workers–recipient workers = 1:10) was observed to be 4.33%, 15%, and 16.33% at 48 h post-treatment, with concentrations of 0.025%, 0.05%, and 0.10%, respectively. The secondary mortalities increased to 11%, 26%, and 37.67% at 72 h post-treatment (Figure 5E).

## 3. Discussion

In the present study, we conducted a series of tests to assess the contact toxicity of azadirachtin, celangulins, and veratramine on red imported fire ants. The results indicated that the LD_50_ (lethal dose 50%) values of azadirachtin, celangulin, and veratramine were 0.200, 0.046, and 544.610 ng/ant, respectively, suggesting that RIFAs exhibit greater sensitivity to azadirachtin and celangulin compared to matrine, rotenone, and pyrethrin, with LD_50_ values of 0.24, 50.929, and 13.590 ng/ant, respectively, 24 h after treatment [60]. For celangulin, Qi et al. [28] demonstrated that the LD50 values for celangulin in birds (quails) exceeded 2800 mg/kg over a 72 h period. This substantiates the notion that, when celangulin is employed for pest control in field settings, it is likely to offer a safe profile for mammals such as birds. Yet, veratramine appeared to have an ineffective characteristic compared to the other tested botanicals when considering their impact on ants. This result implies that azadirachtin and celangulins hold significant potential as toxicants that can induce adverse effects on the invasive ant RIFA.

The term “knockdown” in insects refers to the state of intoxication and partial paralysis that typically precedes death following the application of an insecticide [61]. In our investigation, we determined the median knockdown time (KT_50_) and KT_95_ values of azadirachtin to be 14.581 and 30.493 min, respectively, at a concentration of 50 mg/L on larger-size workers of RIFA. Moreover, our results revealed that celangulin yielded a KT_95_ value of 15.287 min for workers of RIFA, thereby exhibiting a quicker knockdown time in comparison to bifenthrin, a chemical pesticide employed at a concentration of 20 mg/L (KT_95_, 16.611 min), although it was slower than dimefluthrin, a chemical pesticide used at a concentration of 5 mg/L (KT_95_, 2.825 min) [62]. However, veratramine did not exhibit any knockdown effect on large worker ants within one hour, even at a high concentration of 500 mg/L.

The lethal effects of chemical toxicants on pest ants have been extensively studied [63,64]. For example, Buczkowski et al. [64] evaluated the median lethal time (LT_50_) of Argentine ants treated with a professional concentration application of mixed bifenthrin (8.74%), permethrin (35.1%), and fipronil (10.1%) on concrete. The LT_50_ value for this treatment was 7 h. For RIFA, the LT_50_ values were 40.8, 246.6, and 319.6 min when treated with bifenthrin (0.06%), fipronil (0.06%), and chlorfenapyr (0.5%), respectively [64], but there is limited research on botanical toxicants. Our results demonstrated a negative correlation between the concentrations of azadirachtin and celangulin and their lethal times. Celangulins showed higher toxicity compared to azadirachtin and veratramine. When drip treated with 0.125 mg/L azadirachtin, the LT_50_ and LT_95_ values were 60.410 and 403.803 h, respectively. For 0.125 mg/L celangulin, the LT_50_ and LT_95_ values were 9.905 and 25.227 h, respectively. Lastly, for 200 mg/L veratramine, the LT_50_ and LT_95_ values were 46.967 and 169.399 h, respectively. In addition, compared to azadirachtin and celangulin, veratramine showed a slower lethal time with an LT_50_ of 152.972 h and an LT_95_ of 588.644 h after being treated with soil contaminated with a concentration of 8 μg/g. Interestingly, in the case of RIFA, a higher donor-to-recipient workers ratio did not result in a significantly faster lethal time when treated with high concentrations (500 mg/L) of azadirachtin. The first-round toxicity transmission demonstrated an LT_50_ of 6.139 h and an LT_95_ of 11.729 h for a ratio of 1:10, and an LT_50_ of 5.113 h and an LT_95_ of 12.294 h for a ratio of 1:5. Moreover, in the case of dust treatment, celangulin exhibited a slower lethal time compared to azadirachtin. The LT_50_ and LT_95_ values for celangulin were 37.435 h and 79.107 h, respectively, while for azadirachtin, the LT_50_ was 5.769 h and the LT_95_ was 11.947 h after being treated with a 0.10% dust formulation. However, it is important to note that there is considerable interspecific variation in ant susceptibility to broad-spectrum insecticide chemistries [64]. This indicates that such variation may also exist among botanical toxicants. Therefore, future studies should test more ant species to confirm this hypothesis.

The determination of lethal concentrations is crucial for verifying the minimum residues required to maintain efficacy [65]. These concentrations can then be used to establish minimum residue levels for nontarget insects. A previous study reported that the LC_50_ values for the synthetic pesticides bifenthrin and tefluthrin in potting soil, when tested on female alates of RIFA, were 1.1 mg/L and 8.5 mg/L, respectively. The LC_95_ values for bifenthrin and tefluthrin were 5.2 mg/L and 19.0 mg/L, respectively [65]. In our study, we observed that soil contaminated with 8 μg/g and 4 μg/g of azadirachtin and celangulin, respectively, had a relatively high lethal effect on fire-ant workers. However, when workers were exposed to lower concentrations (below 2 μg/g for azadirachtin and 1.6 μg/g for celangulin), they exhibited lower sensitivity with low mortalities (<17%). Veratramine, even at a high concentration of 100 μg/g, did not cause a significant lethal effect on RIFA (mortality < 7.5%).

To mitigate the detrimental impact of pathogen and parasite transmission within social insect colonies, the removal and disposal of waste and deceased colony members from the nest area and their subsequent placement on a dump site is one of the most crucial defense mechanisms [66,67]. This process directly or indirectly influences individual and social interactions among colony members. Insecticides that possess the ability of horizontal transfer, which is a significant mode of action, have been utilized to facilitate this function in social insects through the behavior of replacing the corpses of colony members [68,69]. A study reported that red imported fire ants, being a social insect, were found to be unaffected by the presence of corpses treated with any of the test chemicals, indicating that they were not deterred from removing them from the nest area [70]. However, untreated ants carrying and moving corpses killed by certain insecticides may acquire a lethal dose of the toxicants [67,70]. In the case of synthetic pesticides, Zhang et al. [71] reported that a significant horizontal transfer effect was achieved in the first-round transmission (mortality > 65% for 108 h) when recipient ants were mixed with donor ants previously exposed to 10 μg/cm^2^ of cycloxaprid or imidacloprid. Similarly, bifenthrin demonstrated significant horizontal activity, with 95% of the recipient ants killed at 20 °C when 20% of the ants were treated (donors–recipients ratio = 1:4), and 60% mortality at 20 °C when 10% were treated (donors–recipients ratio = 1:9), and 30 °C when 20% were treated [70]. In the present study, it was observed that azadirachtin demonstrated significantly faster contact toxicity activity and a higher horizontal transfer effect among RIFAs compared to celangulin and veratramine. The increased mortality observed with azadirachtin may be attributed to enhanced contact and transfer between worker ants. After 48 h of treatment with 500 mg/L, 250 mg/L, and 100 mg/L of azadirachtin, the secondary mortality (mortality of recipient workers) (donors–recipients ratio = 1:10) was 100%, 100%, and 85%, respectively. The tertiary mortalities (mortality of secondary recipient workers) were both higher than 61% at 48 h when treated with 500 mg/L and 250 mg/L of azadirachtin. In contrast, although the secondary mortality of recipient workers was 100% and 89.45% when treated with 1000 mg/L and 500 mg/L of celangulin, respectively; the effectiveness of celangulin in inducing tertiary mortalities (donors: recipients = 1:10 and 1:5) was ineffective, with both values being lower than 4% after 48 h of treatment with 1000 mg/L of celangulin.

Similarly, the dust formulation of azadirachtin demonstrated strong horizontal toxicity transfer in the secondary mortality of fire-ant recipients. After 24 h of treatment with 0.05% and 0.10% azadirachtin dust, the secondary mortality of recipient workers (donors–recipients = 1:10) was 96.67% and 99.67%, respectively. The tertiary mortality was over 31% and 43% after treatment with 0.10% azadirachtin dust for 48 and 72 h, respectively. In contrast, for celangulin dust, although the secondary mortalities of recipients were over 93%, it was found to be ineffective in the second-round transmission (tertiary mortalities < 18%) after treatment with 0.10% concentration dust. Based on the tested results mentioned above, the transmission effect of the second round of 0.05% azadirachtin dust appeared to be greater than that of 1.0% rotenone and 0.3% pyrethrin dust, which exhibited tertiary mortalities of only 7.75% and 18.5%, respectively, but inferior to that of 1.0% matrine dust (49.5%) after 48 h [60]. Considering that azadirachtin and celangulin are toxic and can lead to a higher number of corpses within the nest, these compounds can result in a higher horizontal transfer rate among worker ants. This effect can be further amplified when there is a higher donor–recipient worker ratio, accelerating and increasing the deaths of recipient ants.

Additionally, it is important to acknowledge that the application of botanical toxicants may have a significant impact on the interaction between invasive ants and native ants, specifically in terms of competitive behavior [72]. When invasive ants that were exposed to these toxicants interacted with nonexposed native ants, they exhibited lightened aggression and a decreased likelihood of survival. On the other hand, nonexposed invasive ants displayed heightened aggression and increased probability of survival when interacting with exposed native ants. Hence, the exposure of native species to azadirachtin and celangulin, unintentional to their target, could potentially enhance or diminish their survival chances based on the exposure status of the invasive species. Considering that different species within a community have distinct food-resources preferences and, consequently, varying levels of pesticide exposure, nontarget exposure has the potential to alter community dynamics and influence the success of invasion.

Yet it is worth noting that the doses employed in the present investigation may surpass those realistically encountered by nontarget insects in natural environments. Therefore, it is imperative to conduct further evaluations on the residual concentrations of azadirachtin and celangulin when these substances are used in the field to obtain concentrations that are more representative of real-world conditions.

In summary, the results of this study provide compelling evidence to support the potential efficacy of azadirachtin and celangulin as novel approaches for the management of *S. invicta*. These findings highlight the promising prospects of utilizing these natural plant pesticides in the control and mitigation of *S. invicta* infestations. To reinforce these conclusions, further studies should be conducted using field-realistic concentrations of azadirachtin and celangulin. Furthermore, the toxicity of these botanical toxicants against native ants will be further evaluated under controlled laboratory conditions and in conjunction with field trials.

## 4. Conclusions

In this study, we evaluated the contact toxicity, knockdown time, lethal time, and toxicity transmission of three natural plant pesticides (azadirachtin, celangulin, and veratramine) on red imported fire ants, *S. invicta*, a notorious invasive species. Our research findings indicated that azadirachtin and celangulin exhibited relatively high toxicity. Additionally, azadirachtin and celangulin were found to exhibit high horizontal transfer among RIFAs, with high secondary mortalities and tertiary mortalities, as well as with their dust formulations. However, veratramine did not exhibit significant toxicity or toxicity transfer effects on RIFAs, even at high concentrations. These findings suggest that azadirachtin and celangulin have a highly prominent potential in controlling RIFAs.

## 5. Materials and Methods

### 5.1. Insects and Experimental Materials

The study employed commercially available products of botanical pesticides, including 0.5% azadirachtin soluble concentrate SL (Yunnan Lvrong Biological Industry Development Co., Ltd., Kunming, China), 1% celangulin soluble concentrate SL (Shandong Huimin Zhonglian Biotechnology Co., Ltd., Jinan, China), and 0.5% veratramine soluble concentrate SL (Yangling Fuji Biotechnology Co., Ltd., Xianyang, China). The use of these products was permitted on organic farms. The experiments utilized Arenosol (Senmino Horticulture, Guangzhou, China), activated clay (Henan Hongshu Environmental Protection Material, Zhengzhou, China), and talc powder (Zhengzhou Hongsheng Talc Powder Products, Zhengzhou, China). The fire-ant colonies were collected from the Huadu district of Guangzhou, Guangdong Province (113°2′16″ E, 23°27′37″ N) and reared in plastic boxes with inner walls coated with a mixture of talc powder and ethanol to prevent escape [73]. The ant colonies were provided with yellow mealworm *Tenebrio molitor* and 10% *w*/*w* sugar water as food. All colonies were maintained under laboratory conditions of 26 °C, a 14:10 (L:D) cycle, and a relative humidity of 60% for two weeks before the experiments.

### 5.2. Contact Toxicity of Azadirachtin, Celangulin, and Veratramine on Workers

For the experiments, large worker ants with a consistent body-size range of 1.20–1.40 mm head width were carefully selected. The three botanical pesticides, namely azadirachtin, celangulin, and veratramine, were diluted with acetone. Using the method described by Lei Wang [74], one microliter of each concentration solution was meticulously applied to the pronotum of the selected workers. Subsequently, the treated workers were placed in plastic bowls with a diameter of 10 mL and provided with 10% *w*/*w* sugar water. The number of deceased workers was recorded at specific time intervals of 12, 24, 36, and 48 h post-treatment. Various concentrations were tested for each botanical pesticide. Azadirachtin concentrations of 1, 0.5, 0.25, 0.125, and 0.1 mg/L were used. Celangulin concentrations of 0.13, 0.0625, 0.042, 0.031, and 0.025 mg/L were tested. Veratramine concentrations of 500, 200, 100, 50, and 25 mg/L were examined. Acetone was employed as the control. Each concentration was replicated three times, with 20 large workers used for each replicate. The entire experiment was conducted twice, utilizing two different ant colonies. To prevent rapid volatilization of the acetone solvent, all solutions were consistently kept on ice throughout the entire experiment. All bioassays were performed under laboratory conditions of 26 °C, a 14:10 (L:D) cycle, and 60% relative humidity.

### 5.3. Knockdown Efficiency of Azadirachtin, Celangulin, Veratramine on Workers

To evaluate the knockdown efficacy of the three botanical pesticides, large workers were carefully placed in a glass Petri dish (90 mm) with a rim coated with fulon to prevent their escape. Subsequently, a solution containing the botanical pesticides was sprayed onto the fire-ant workers using a hand-held spray bottle, with a total volume of 0.2 mL administered through two sprays [70]. Different doses of azadirachtin, celangulin, and veratramine were tested. Azadirachtin doses of 500, 100, 50, and 10 mg/L were utilized. Celangulin doses of 500, 100, 50, and 10 mg/L were examined. Veratramine doses of 500 and 100 mg/L were tested. Acetone, which was used as a solvent for diluting the botanical pesticides, served as the control. The number of workers unable to maintain their balance and exhibiting shaking movements was recorded at regular intervals for a duration of 1 min until almost all tested workers had fallen. Each treatment was replicated three times, with 50 large workers used for each replicate. The entire experiment was conducted twice, utilizing two different ant colonies.

### 5.4. Effect of Soil Contaminated with Azadirachtin, Celangulin, and Veratramine on Workers

To assess the impact of soil contamination by azadirachtin, celangulin, and veratramine on RIFA, we conducted an experiment to evaluate the lethal effect of these botanicals on fire ant workers under laboratory conditions. For this experiment, solutions of the three botanical pesticides were mixed thoroughly with arenosol to obtain the test concentrations of toxicity, of all which were tested after drying for 24 h (DHG Series Heating and Drying Oven, 40 °C, Shanghai Qixin Scientific Instrument Co., Ltd., Shanghai, China). Azadirachtin doses of 8, 4, 2, and 0.8, 1.6 μg/g toxic soil were tested; celangulin doses of 8, 4, 1.6, and 0.8 μg/g were tested; and veratramine doses of 200, 100, 40, and 20 μg/g were tested. Purified water served as the control treatment. Each treatment was replicated three times, and the entire experiment was conducted twice using two different ant colonies. The mortality of the workers was recorded at 6, 12, 24, 36, 48, 60, 72, 84, and 96 h post-treatment.

### 5.5. Horizontal Toxicity Transfer of Azadirachtin, Celangulin, Veratramine on Workers

This study aims to evaluate the horizontal transfer efficiency of azadirachtin, celangulin, and veratramine among fire-ant workers using both solution and toxic dust formulations. The tested solutions were prepared by diluting azadirachtin, celangulin, and veratramine with a solution of acetone and purified water (8:2) at varying concentrations. Control treatments were prepared using purified water with acetone. To prevent rapid acetone evaporation, all solutions were kept on ice during the experiment. The horizontal transfer experiment followed the procedure described by Buczkowski [75] and was divided into two parts, the first and second rounds of horizontal transfer. In the first round, test solutions were dripped onto the pronotum of large workers, and the treated large workers (donors) were placed in a plastic bowl containing healthy large workers (recipients) from the same colony at different ratios. The recipients’ mortality (secondary mortality) was recorded at different time intervals post-treatment. In the second round, recipients’ corpses from the first-round trial were used as secondary donors, and their mortality (tertiary mortality) was recorded when placed in a plastic bowl containing secondary recipients. For toxic dust testing, azadirachtin, celangulin, and veratramine were mixed with activated clay and talc powder to obtain test concentrations. Three replicates were conducted for each treatment, and the entire experiment was repeated twice using different colonies. All bioassays were conducted under laboratory conditions of 25 °C with a 14 h L:10 h D cycle and a relative humidity of 60%.

### 5.6. Data Analysis

The normality of the data was assessed using the one-sample Kolmogorov–Smirnov test, while Levene’s test was employed to evaluate the homogeneity of variance. In cases where the data exhibited homogeneous variance, a one-way ANOVA and a Tukey’s test were used for multiple comparisons. For data with uneven variance but meeting the conditions of normal distribution, Tamhane’s test was applied for multiple comparisons. Non-normally distributed data were analyzed using the nonparametric Kruskal–Wallis test to compare medians. Data processing was conducted using Excel 2013 and SPSS 21.0 software, and the laboratory experimental data for two colonies were collectively analyzed using pooled SPSS 21.0 data. The LD_25_, LD_50_, LD_95_, KT_25_, KT_50_, KT_95_, LT_25_, LT_50_, and LT_90_ values of the botanical toxicants were determined using Probit in SPSS 21.0.

## Figures and Tables

**Table 1:** Lethal dose (LD) toxicity of azadirachtin, celangulin, and veratramine on red imported fire ants.

Pesticides	Numbers of Workers	Time after Treatment (hours)	Regression Equation ^(1)^	LD_25_ (95% CI) (ng/ant)	LD_50_ (95% CI) (ng/ant)	LD_95_ (95% CI) (ng/ant)	Standard Error	*χ* ^2^	*df*	*p*-Value
Azadirachtin	120	12	*Y* = 2.615 *X* + 1.132	0.204 (0.179~0.228)	0.369 (0.332~0.413)	1.571 (1.265~2.071)	0.180	23.501	28	0.708
		24	*Y* = 3.396 *X* + 2.375	0.126 (0.113~0.140)	0.200 (0.183~0.218)	0.610 (0.520~0.746)	0.238	25.944	28	0.576
		36	*Y* = 3.639 *X* + 2.892	0.105 (0.093~0.116)	0.160 (0.147~0.174)	0.454 (0.390~0.556)	0.284	26.697	28	0.535
		48	*Y* = 3.968 *X* + 3.275	0.101 (0.090~0.111)	0.149 (0.138~0.162)	0.388 (0.335~0.472)	0.326	25.900	28	0.579
Celangulin	120	12	*Y* = 2.685 *X* + 2.866	0.048 (0.042~0.054)	0.086 (0.075~0.102)	0.351 (0.250~0.587)	0.244	36.130	28	0.139
		24	*Y* = 3.998 *X* + 5.337	0.031 (0.027~0.035)	0.046 (0.042~0.051)	0.119 (0.098~0.159)	0.304	50.115	28	0.006
		36	*Y* = 3.772 *X* + 5.321	0.026 (0.022~0.029)	0.039 (0.035~0.043)	0.106 (0.087~0.142)	0.316	46.304	28	0.016
		48	*Y* = 3.915 *X* + 5.679	0.024 (0.020~0.027)	0.035 (0.032~0.039)	0.093 (0.077~0.126)	0.342	50.706	28	0.005
Veratramine	120	12	*Y* = 0.460 *X* − 2.547	11,755.790	*	*	0.184	118.27	28	<0.0001
		24	*Y* = 1.348 *X* − 3.688	172.088 (117.776~259.926)	544.610 (340.822~1313.733)	9041.335 (2855.794~108,809.374)	0.149	77.207	28	<0.0001
		36	*Y* = 1.576 *X* − 3.734	87.370 (62.217~114.368)	234.074 (177.387~339.071)	2588.682 (1305.057~8398.921)	0.140	65.041	28	<0.0001
		48	*Y* = 1.632 *X* − 3.598	61.947 (38.885~85.373)	160.470 (118.469~232.609)	1634.820 (823.643~5833.635)	0.137	95.179	28	<0.0001

^(1)^ Probit (*Y*) = intercept + B*X* (covariate *X* is converted by logarithm based 10). CI: confidence interval. *: indicates the numerical value is abnormal.

**Table 2:** The lethal time (LT) of azadirachtin, celangulin, and veratramine of drip treatment on red imported fire ants.

Pesticides	Concentration (mg/L)	Numbers of Workers	Regression Equation ^(1)^	LT_25_ (95% CI) (h)	LT_50_ (95% CI) (h)	LT_95_ (95% CI) (h)	Standard Error	*χ* ^2^	*df*	*p*-Value
Azadirachtin	1	120	*Y* = 5.252 *X* − 4.659	5.736 (2.068~7.744)	7.709 (4.001~9.440)	15.856 (13.993~21.868)	1.494	3.199	22	1.000
	0.5	120	*Y* = 2.917 *X* − 2.740	5.105 (2.637~7.261)	8.693 (5.689~11.049)	31.841 (26.307~43.379)	0.382	31.130	22	0.093
	0.25	120	*Y* = 1.915 *X* − 2.349	7.485 (4.573~10.031)	16.839 (13.430~19.739)	121.642 (84.794~225.448)	0.269	17.383	22	0.742
	0.125	120	*Y* = 1.994 *X* − 3.551	27.719 (22.285~33.394)	60.410 (46.981~100.087)	403.803 (191.132~2204.853)	0.319	30.909	22	0.098
Celangulin	0.125	120	*Y* = 4.051 *X* − 4.035	6.751 (4.010~8.820)	9.905 (7.099~11.970)	25.227 (21.105~34.141)	0.493	43.276	22	0.004
	0.063	120	*Y* = 1.455 *X* − 1.687	4.965 (0.144~10.201)	14.433 (3.681~21.097)	194.821 (81.880~*)	0.263	72.207	22	<0.0001
	0.042	120	*Y* = 2.080 *X* − 2.964	12.615 (9.326~15.326)	26.618 (23.261~30.389)	164.431 (111.778~311.827)	0.272	18.712	22	0.663
	0.031	120	*Y* = 1.925 *X* − 3.334	24.067 (19.879~27.895)	53.918 (44.451~74.239)	385.491 (207.476~1231.782)	0.304	23.493	22	0.374
Veratramine	500	120	*Y* = 3.414 *X* − 4.737	15.486 (11.635~18.541)	24.407 (20.767~28.126)	74.021 (57.031~116.236)	0.305	55.526	22	<0.0001
	200	120	*Y* = 2.952 *X* − 4.936	27.755 (23.432~31.731)	46.967 (40.323~59.841)	169.399 (110.8807~384.383)	0.369	34.883	22	0.04
	100	120	*Y* = 3.106 *X* − 5.434	34.086 (28.339~41.136)	56.202 (45.495~87.904)	190.278 (110.718~730.029)	0.419	52.931	22	<0.0001
	50	120	*Y* = 1.027 *X* − 2.455	54.316 *	246.601 *	9871 *	0.324	102.523	22	<0.0001

^(1)^ Probit (*Y*) = intercept + B*X* (covariate *X* is converted by logarithm based 10). CI: confidence interval. *: indicates the numerical value is abnormal.

**Table 3:** The knockdown time of azadirachtin and celangulin for spray treatment on red imported fire ants.

Pesticides	Concentration (μg mL^−1^)	Numbers of Workers	Regression Equation ^(1)^	KT_25_ (95% CI) (min)	KT_50_ (95% CI) (min)	KT_95_ (95% CI) (min)	Standard Error	*χ* ^2^	*df*	*p*-Value
Azadirachtin	500	300	*Y* = 8.229 *X* − 6.381	4.937 (4.447~5.326)	5.963 (5.554~6.362)	9.449 (8.565~10.945)	0.283	692.822	58	<0.0001
	100	300	*Y* = 6.504 *X* − 6.821	8.811 (8.192~9.357)	11.187 (10.617~11.764)	20.027 (18.422~22.297)	0.157	1123.680	118	<0.0001
	50	300	*Y* = 5.134 *X* − 5.974	10.774 (9.949~11.527)	14.581 (13.734~15.487)	30.493 (27.337~35.098)	0.115	1411.892	136	<0.0001
	10	300	*Y* = 5.362 *X* − 7.383	17.834 (16.911~18.703)	23.825 (22.796~24.958)	48.286 (44.054~54.062)	0.124	973.064	154	<0.0001
Celangulin	500	300	*Y* = 10.238 *X −* 8.690	6.067 (5.836~6.273)	7.061 (6.858~7.264)	10.221 (9.761~10.815)	0.341	215.920	64	<0.0001
	100	300	*Y* = 9.699 *X* − 9.029	7.268 (6.078~8.091)	8.530 (7.578~9.456)	12.605 (11.062~16.221)	0.267	4376.928	88	<0.0001
	50	300	*Y* = 0.442 *X* − 5.110	10.038 (9.783~10.273)	11.565 (11.357~11.772)	15.287 (14.944~15.680)	0.011	291.248	112	<0.0001
	10	300	*Y* = 8.896 *X* − 11.465	16.332 (15.990~16.653)	19.447 (19.144~19.749)	29.768 (28.979~30.669)	0.181	433.508	184	<0.0001

^(1)^ Probit (*Y*) = intercept + B*X* (covariate *X* is converted by logarithm based 10). CI: confidence interval.

## Data Availability

The raw data and materials will be made available by the authors, without undue reservation, to any qualified researchers.

## Appendix A

**Table A1:** The lethal time (LT) of red imported fire ants exposed to soil contaminated with azadirachtin, celangulin, and veratramine.

Pesticides	Concentration (μg/g)	Numbers of Workers	Regression Equation ^(1)^	LT_25_ (95% CI) (h)	LT_50_ (95% CI) (h)	LT_95_ (95% CI) (h)	Standard Error	*χ* ^2^	*df*	*p*-Value
Azadirachtin	8	180	*Y* = 6.054 *X* − 10.081	35.790 (31.574~39.325)	46.256 (42.438~49.963)	86.469 (77.240~101.228)	0.277	254.547	52	<0.0001
	4	180	*Y* = 2.925 *X* − 5.573	47.247 (37.105~56.232)	80.341 (67.077~106.253)	293.234 (185.417~768.755)	0.188	425.508	52	<0.0001
	2	180	*Y* = 1.044 *X* − 3.254	295.386 (159.397~1568.522)	1307.327 (445.387~*)	*	0.182	99.323	52	<0.0001
Celangulin	8	180	*Y* = 5.183 *X* − 7.852	24.258 (22.685~25.718)	32.734 (31.212~34.213)	67.979 (63.988~72.890)	0.245	60.718	52	0.191
	4	180	*Y* = 3.797 *X* − 6.071	26.395 (22.195~30.063)	39.736 (35.525~43.891)	107.753 (91.745~134.662)	0.176	215.904	52	<0.0001
	1.6	180	*Y* = 1.229 *X* − 3.580	230.605 (157.730~463.469)	815.608 (418.612~2896.902)	*	0.199	27.145	52	0.998
veratramine	200	180	*Y* = 2.811 *X* − 6.140	88.030 (65.095~181.985)	152.972 (103.897~1220.205)	588.644 (237.458~*)	0.246	1009.721	58	<0.0001
	100	180	*Y* = 1.830 *X* − 5.191	293.922 (159.568~*)	686.814 (266.745~*)	*	0.366	180.105	58	<0.0001

^(1)^ Probit (*Y*) = intercept + B*X* (covariate *X* is converted by logarithm based 10). CI: confidence interval. *: indicates the numerical value is abnormal.

**Table A2:** The lethal time (LT) of azadirachtin, celangulin, and veratramine in the first round of toxicity transmission on red imported fire ants.

Pesticides	Donor–Recipient Workers Ratio	Concentration (mg/L)	Numbers of Workers	Regression Equation ^(1)^	LT_25_ (95% CI) (h)	LT_50_ (95% CI) (h)	LT_95_ (95% CI) (h)	Standard Error	*χ* ^2^	*df*	*p*-Value
Azadirachtin	1:10	500	180	*Y* = 5.850 *X* − 4.611	4.708 (4.146~5.157)	6.139 (5.678~6.551)	11.729 (10.597~13.544)	0.606	16.369	28	0.960
		250	180	*Y* = 6.387 *X* − 5.211	5.131 (4.638~5.534)	6.544 (6.134~6.939)	11.841 (10.716~13.628)	0.636	16.447	28	0.959
		100	180	*Y* = 1.981 *X* − 2.087	5.164 (1.934~8.122)	11.310 (6.791~15.514)	76.532 (46.132~236.895)	0.145	218.230	28	<0.0001
		50	180	*Y* = 1.292 *X* − 1.618	5.374 (1.057~9.449)	17.882 (10.615~27.400)	335.538 (119.534~9051.041)	0.135	174.437	28	<0.0001
	1:5	500	180	*Y* = 4.317 *X* − 3.059	3.568 (2.797~4.182)	5.113 (4.406~5.680)	12.294 (10.901~14.666)	0.511	19.901	28	0.868
		250	180	*Y* = 6.024 *X* − 4.538	4.378 (3.733~4.854)	5.666 (5.171~6.071)	10.624 (9.535~12.572)	0.763	13.325	28	0.991
		100	180	*Y* = 3.384 *X* − 2.771	4.164 (2.698~5.391)	6.589 (5.004~7.972)	20.177 (16.106~28.980)	0.254	106.407	28	<0.0001
		50	180	*Y* = 2.451 *X* − 2.200	4.193 (2.522~5.742)	7.092 (5.778~9.846)	37.067 (28.264~56.641)	0.170	102.547	28	<0.0001
Celangulin	1: 10	1000	180	*Y* = 3.331 *X* − 3.333	6.282 (4.590~7.772)	10.013 (8.154~11.848)	31.213 (24.772~44.276)	0.198	120.982	28	<0.0001
		500	180	*Y* = 2.190 *X* − 2.503	5.839 (4.006~9.362)	13.900 (10.370~17.515)	78.374 (52.267~162.800)	0.148	148.057	28	<0.0001
		200	180	*Y* = 1.831 *X* − 2.813	14.741 (8.543~20.204)	34.435 (25.214~57.636)	272.635 (121.368~2161.620)	0.153	193.586	28	<0.0001
		100	180	*Y* = 2.438 *X* − 4.420	34.358 (27.642~44.778)	64.961 (48.730~119.402)	307.087 (151.841~1669.586)	0.252	110.416	28	<0.0001
	1: 5	1000	180	*Y* = 2.488 *X* − 2.475	5.295 (2.939~7.392)	9.886 (6.988~12.599)	45.315 (32.263~82.795)	0.163	166.706	28	<0.0001
		500	180	*Y* = 2.654 *X* − 2.449	4.661 (2.817~6.308)	8.369 (6.150~10.405)	34.872 (26.266~55.458)	0.179	127.411	28	<0.0001
		200	180	*Y* = 2.329 *X* − 3.178	11.877 (9.177~14.324)	23.135 (19.697~17.338)	117.601 (83.002~202.065)	0.156	80.696	28	<0.0001
		100	180	*Y* = 1.823 *X* − 3.381	30.499 (21.404~47.992)	71.484 (46.099~249.272)	570.578 (188.998~*)	0.194	172.852	28	<0.0001
Veratramine	1: 5	500	180	*Y* = 2.603 *X* − 5.408	65.907 (49.526~171.041)	119.699 (74.052~728.873)	512.977 (187.419~*)	0.489	67.159	28	<0.0001
		250	180	*Y* = 2.358 *X* − 5.036	70.701 (48.958~463.588)	136.587 (74.325~4712.848)	680.498 (193.043~*)	0.451	94.576	28	<0.0001

^(1)^ Probit (*Y*) = intercept + B*X* (covariate *X* is converted by logarithm based 10). CI: confidence interval. *: indicates the numerical value is abnormal.

**Table A3:** The lethal time (LT) of azadirachtin, celangulin, and veratramine in the second round of toxicity transmission on red imported fire ants.

Pesticides	Donor–Recipient Workers Ratio	Concentration (mg/L)	Numbers of Workers	Regression Equation ^(1)^	LT_25_ (95% CI) (h)	LT_50_ (95% CI) (h)	LT_95_ (95% CI) (h)	Standard Error	*χ* ^2^	*df*	*p*-Value
Azadirachtin	1:10	500	180	*Y* = 1.328 *X* − 1.783	6.834 (0.420~12.660)	22.012 (11.347~48.572)	381.461 (107.220~ *)	0.137	311.337	28	<0.0001
		250	180	*Y* = 1.246 *X* − 1.829	8.453 (0.684~14.924)	29.403 (17.232~109.090)	614.665 (140.090~ *)	0.142	259.372	28	<0.0001
	1:5	500	180	*Y* = 1.218 *X* − 1.787	8.190 (0.467~14.821)	29.311 (16.763~111.454)	656.849 (144.570~ *)	0.138	281.841	28	<0.0001
		250	180	*Y* = 1.024 *X* − 1.675	9.477 (0.033~18.017)	43.169 (23.041~5991.40)	1742.010 (214.271~ *)	0.139	272.476	28	<0.0001

^(1)^ Probit (*Y*)= intercept + B*X* (covariate *X* is converted by logarithm based 10). CI: confidence interval. *: indicates the numerical value is abnormal.

**Table A4:** The lethal time (LT) of azadirachtin, celangulin, and veratramine dust in the first round of toxicity transmission on red imported fire ants.

Pesticides	Donor–Recipient Workers Ratio	Concentration	Numbers of Workers	Regression Equation ^(1)^	LT_25_ (95% CI) (h)	LT_50_ (95% CI) (h)	LT_95_ (95% CI) (h)	Standard Error	*χ* ^2^	*df*	*p*-Value
Azadirachtin	1:10	0.10%	300	*Y* = 5.203 *X* − 3.960	4.281 (3.799~4.683)	5.769 (5.361~6.130)	11.947 (10.957~13.392)	0.436	42.549	40	0.362
		0.05%	300	*Y* = 4.012 *X* − 3.814	6.061 (5.572~6.521)	8.926 (8.407~9.442)	22.941 (21.082~25.317)	0.197	34.073	40	0.733
		0.25%	300	*Y* = 2.237 *X* − 3.608	20.480 (15.402~25.023)	41.007 (34.029~50.118)	222.950 (146.464~451.283)	0.106	310.347	40	<0.0001
Celangulin	1:10	0.10%	300	*Y* = 5.062 *X* − 7.964	27.544 (23.732~30.717)	37.435 (33.943~40.900)	79.107 (68.614~97.342)	0.211	267.649	40	<0.0001
		0.05%	300	*Y* = 5.762 *X* − 9.329	31.769 (26.781~35.684)	41.596 (37.237~46.006)	80.263 (68.566~103.721)	0.243	421.620	40	<0.0001
		0.25%	300	*Y* = 5.745 *X* − 9.325	32.034 *	41.977 *	81.155 *	0.239	33,593.762	40	<0.0001
veratramine	1:10	0.10%	300	*Y* = 1.886 *X* − 4.231	76.925 (58.661~139.229)	175.298 (108.051~648.344)	1306.511 (429.367~*)	0.184	240.621	40	<0.0001

^(1)^ Probit (*Y*) = intercept + B*X* (covariate *X* is converted by logarithm based 10). CI: confidence interval. *: indicates the numerical value is abnormal.

**Table A5:** The lethal time (LT) of azadirachtin, celangulin, and veratramine dust in the second round of toxicity transmission on red imported fire ants.

Pesticides	Donor–Recipient Workers Ratio	Concentration	Numbers of Workers	Regression Equation ^(1)^	LT_25_ (95% CI) (h)	LT_50_ (95% CI) (h)	LT_95_ (95% CI) (h)	Standard Error	*χ* ^2^	*df*	*p*-Value
Azadirachtin	1:10	0.10%	300	*Y* = 1.641 *X* − 3.221	35.624 (30.429~40.838)	91.773 (74.724~126.388)	922.427 (481.641~2756.992)	0.143	72.877	34	<0.0001
		0.05%	300	*Y* = 1.956 *X* − 3.822	40.660 (35.470~46.229)	89.958 (74.464~120.487)	623.843 (357.659~1582.785)	0.157	84.158	34	<0.0001
		0.25%	300	*Y* = 1.823 *X* − 3.734	47.668 (42.561~53.932)	111.736 (91.078~152.414)	892.078 (501.760~2228.251)	0.163	54.778	34	0.013

^(1)^ Probit (*Y*) = intercept + B*X* (covariate *X* is converted by logarithm based 10). CI: confidence interval.
